# Did animal offer relevant model for Bevacizumab testing?

**DOI:** 10.1038/sj.bjc.6604693

**Published:** 2008-10-07

**Authors:** C Eveno, S Gaujoux, G Tobelem, M Pocard

**Affiliations:** 1 Unité Inserm U689, équipe ‘angiogenèse et cibles thérapeutiques’, hôpital Lariboisière, Institut des Vaisseaux et du Sang, 8, rue Guy-Patin, Paris, cedex 10 75475, France; 2 Département médico-chirurgical de pathologie digestive, hôpital Lariboisière, 2, rue Ambroise-Paré, Paris, cedex 10 75475, France; 3 Université Paris-Diderot, Paris 7, France

**Sir,**

We have read with great interest the article by Bozec *et al* (2008). In their study, they evaluated on an orthotopic xenograft model, the antitumour efficacy of bevacizumab, erlotinib and irradiation, alone and in combination, on a vascular endothelial growth factor (VEGF) -secreting human head and neck tumour cell line (CAL33). They reported a significant primary tumour mass decrease with drug association but not with bevacizumab alone. And the authors concluded that the efficacy of the combination of bevacizumab, erlotinib and RT might be of clinical importance in the management of head and neck cancer patients.

This work prompted us to analyse the murine model pertinence. We tested human endothelial cell proliferation in the presence of murine or human VEGF. We noticed a characteristic bell-shaped dose–response curve for both human and murine VEGF in the absence of bevacizumab (Figure 1). In the presence of the most efficient concentration of VEGF (12.5 *μ*g ml^−1^), we observed a difference of bevacizumab inhibition between murine and human VEGF-induced proliferation (Figure 2). The endothelial cell proliferation with human VEGF was more inhibited when compared with murine VEGF (with 35 *vs* 17% of decrease).

Several reasons can explain the inefficacy of bevacizumab when tested alone to inhibit human tumour progression in a xenograft mice model: (i) increasing evidences (Liang *et al*, 2006; Yu *et al*, 2008) show that bevacizumab fails to neutralise efficiently murine VEGF because of a weak interaction; (ii) VEGF in sufficient amounts to promote tumour angiogenesis originates from various host cells in the body such as platelets, muscle cells, tumour-associated stromal cells, and in scar (Kerbel, 2008); (iii) murine VEGF is efficient enough to promote human cell growth.

In our opinion, animal models should not be used to conclude on the clinical pertinence of bevacizumab, unless animals express a humanised form of VEGF (Gerber *et al*, 2007).

## Figures and Tables

**Figure 1:**
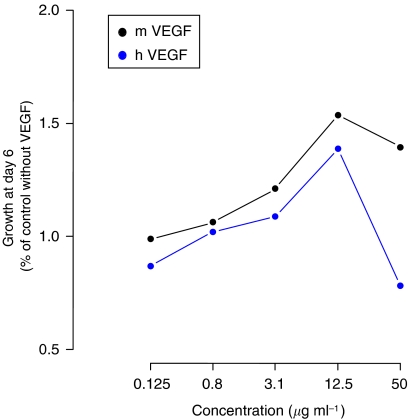
Endothelial cell proliferation assay: HUVECs (human umbilical vein endothelial cells) were incubated with increasing concentrations of h-VEGF (human) or m-VEGF (murine).

**Figure 2:**
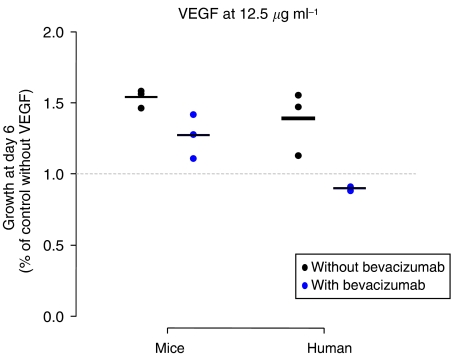
Endothelial cell proliferation assay: HUVECs (human umbilical veinous endothelial cells) were incubated with h-VEGF or m-VEGF (12.5 *μ*g ml^−1^), without and with Bevacizumab.
